# Perception of Old Age by the Inhabitants of Poland

**DOI:** 10.3390/ijerph17072389

**Published:** 2020-04-01

**Authors:** Mariusz Wysokiński, Wiesław Fidecki, Tomasz Plech, Irena Wrońska, Magda Kamila Pawelec, Beata Dziedzic

**Affiliations:** 1 Department of Basic Nursing and Medical Teaching, Chair of Development in Nursing, Faculty of Health Sciences, Medical University of Lublin, 20-081 Lublin, Poland; wieslaw.fidecki@umlub.pl (W.F.); wronska.irena@gmail.com (I.W.); pawelec.magda@wp.pl (M.K.P.); 2 Department of Pharmacology, Faculty of Health Sciences, Medical University of Lublin, Chodźki 4a, 20-093 Lublin, Poland; tomasz.plech@umlub.pl; 3 Department of Development of Nursing and Social & Medical Sciences, Medical University of Warsaw, 02-091 Warsaw, Poland; beata.dziedzic@poczta.onet.pl

**Keywords:** old age, perceiving old age, self-esteem

## Abstract

*Introduction*: People’s self-esteem and public perception of senior citizens both play important roles in perceiving old age. The public perception manifests itself in adopting specific attitudes toward the elderly. *Aim of the work*: The work aimed at attempting to specify how adults and the elderly perceive old age. *Material and method*: The diagnostic poll method was employed as the main research tool, whereas the Rosenberg Self-Esteem Questionnaire by Morris Rosenberg and the Kogan’s Attitudes toward Old People Scale, as well as authors’ own sociodemographic variables metrics, were used as research tools. The investigation was administered in a cohort of 206 people living in Poland. *Results*: The average number of points on the Rosenberg Self-Esteem Questionnaire by Morris Rosenberg (SES) was 29.01 (SD = 4.24). People over the age of 60 (M = 30.07 points), males (M = 32.05 points), those in a relationship (M = 30.22 points), declaring higher education (M = 30.33 points), and a good material situation (M = 30.12 points) enjoyed higher self-esteem. The average number of points on the Kogan’s Attitudes toward Old People Scale (KAOP) in the research cohort was 126.48. The assessment of the elderly was higher among those below 60 (M = 127.06), females (M = 127.29), those in a relationship (M = 129.78), those declaring higher education (M = 128.56), and those in a good material situation (M = 126.99). *Conclusions*: Respondents perceived old age positively, albeit at a low level. It is necessary to review activities undertaken in the sphere of social policy in Poland because activities undertaken to date are failing to improve old age perception. Actions need to be undertaken aiming at raising self-esteem level in Polish senior citizens, and available financial, social, and psychological resources from the government and community associations should all be used to this end.

## 1. Introduction

Old age and the related process of growing old concerns every person. In 2017, the number of people over 65 years of age exceeded the number of children below 5 years of age [1]. According to demographic forecasts for Poland, the percentage of people over 60 is going to increase sharply after 2035 and is expected to reach 40.4% in 2050 [2,3]. Poland will then become the fastest growing old country in Europe. In comparison, there will be 30% of people over 60 in Europe in 2050 [1,2]. Therefore, improving the image of an elderly person and the fostering acceptance of old age by the public, as well as natural results of changing proportions between generations [4], were introduced as priority goals in the document “Polish social policy for the elderly 2030. Safety–Participation–Solidarity.” Thus, shaping a positive perception of old age in the society [5] is one of the major tasks for local communities, as well as for the activities to be undertaken by the Polish government. The individual dimension, e.g., self-esteem, and the communal dimension, e.g., the perception of seniors in the society, both play significant roles in perceiving old age. They both lead to shaping specific attitudes toward seniors. These attitudes may range from negative, e.g., gerontophobia or ageism, to positive, e.g., respect, admiration for experience, wisdom, kindness, dependability, affluence, political power, freedom, eternal youth, and happiness [6,7,8,9,10]. Importantly, self-esteem is considered to be the factor with the greatest influence on the mental sphere of the elderly [11], as self-esteem tends to decrease in old age [12]. According to the research administered in Poland in 2007, elderly people face varied attitudes, however, negative ones seem to predominate in most communities (except the next of kin, neighbors, and local parishes) [13]. This approach is frequently triggered by stereotypical perceptions of old age. Unfortunately, there are no reports on attitudes toward old age among adult and elderly people in various stages of their lives. 

### Aim of the Work

The work aimed at attempting to specify how adult and elderly people perceive old age.

## 2. Material and Method 

The diagnostic poll method was employed as the main research method, whereas the Rosenberg Self-Esteem Questionnaire by Morris Rosenberg, the Kogan’s Attitudes toward Old People Scale, and the authors’ own sociodemographic variables metrics were used as research tools. The Rosenberg Self-Esteem Questionnaire by Morris Rosenberg (SES) scale includes 10 statements that allow the measurement a general level of self-esteem, which includes self-acceptance and the way one perceives oneself. Answers are provided on the four-level range scale, ranging from “I definitely agree” to “I definitely disagree.” The number of points one can score ranges from 10 to 40. The following interpretation of scores was adopted: 10–27 points—low self-esteem, 28–32 points—average or common self-esteem, and 33–40—high self-esteem [6]. Cornabch’s α coefficient for the scale ranged between 0.81 and 0.83 depending on the age group [14]. 

The Kogan’s Attitudes toward Old People Scale (KAOP) questionnaire consists of 34 statements, 17 of which (even ones) are positive and the further 17 (odd ones) are negative. Statements refer to specific fields, such as the place of abode, a diversification of the needs, individual features, intergenerational relationships, dependency, cognitive functioning, one’s looks, and one’s potential. Each statement is described on the Likert’s scale, ranging from “I definitely disagree” to “I definitely agree.” The general range of results for KAOP varies from 34 to 204 points, and higher results represent more positive attitudes. The following point division has been adopted: Negative attitude—34 to 118 points; positive attitude—low level, 119 to 146 points, medium level, 147 to 174 points, and high level, 175 to 204 points [6]. Cornbach’s α coefficient for the KAOP questionnaire was 0.81 [15,16]. 

The absence of chronic somatic and psychiatric diseases (based on medical documentation), not having any medical education (graduating from medical faculties at universities or colleges), and being over the age of 18 were all the criteria for being included into the research cohort. 

The research material was statistically analyzed by means of the statistical IBM SPSS Statistics package. Numerical strength and proportion were provided for categories of answers to specific questions. Quantity variables were described by means of the median and standard deviation, as well as the minimum and the maximum value. Two independent groups were compared by means of the Mann–Whitney test, whereas the ANOVA range Kruskal–Wallis test was used to compare more than two independent groups. The r Pearson correlation coefficient was used to establish a relationship between the variables. Research analysis findings were accepted as statistically significant at the significance level of *p* < 0.05. 

The investigation was administered in compliance with the Helsinki declaration. 

The research was done from March to May 2018 in a cohort of 206 people. It was administered in the lubelskie voivodeship, which is the third largest Polish voivodeship with the area of 25,122 km^2^. However, it is inhabited by merely 5.5% of the total population of the country. Therefore, it is a heavily depopulated area, and the demographic burden index (the number of people at the nonproductive age for 100 people at the productive age) is 65. It is also one of the Polish regions with the greatest share of elderly people in the general population, especially the share of people at the post-productive age (30.5%) [5].

## 3. Results

Respondents’ average age was 47.43 (SD = 19.85). People below the age of 60 predominated (66.01%), as well as females (71.84%), those in a relationship (56.80%), those with higher education (45.15%), and those who judged their material situation as good (90.29%). Table 1 presents a characteristic of the investigated group. 

The first research stage consisted in assessing self-esteem on the basis of the M. Rosenberg’s SES scale. The average number of points was 29.01 (SD = 4.23). This showed self-esteem to be at an average, or common, level. The lowest number of points respondents scored was 17, whereas the highest was 40 points. People bellow 60 years, male, married, with higher education, and good material status presented (or showed, or demonstrated) higher self-esteem.

A statistical difference in self-esteem was discovered in respect of respondent’s sex and material situation. The findings are presented in Table 2. 

The second research stage consisted in assessing people’s attitudes toward the elderly on the basis of the KAOP scale. The average number of points within the research cohort was 126.48. This proved them to have had a positive attitude, albeit at a low level. The attitudes toward old people were more positive for those below 60 (M = 127.06), for females (M = 127.29), for married people (M = 129.78), those declaring higher education (M = 128.56), and a good material situation (M = 126.99). The statistical difference in the KAOP score occurred only with respect to their material situation (Table 3). 

A positive correlation was found within the research cohort between self-esteem and attitudes toward elderly people on the basis of r Pearson’s correlation (r = 0.252; *p* < 0.001). A higher level of self-esteem correlated with a more positive attitude toward elderly people. 

## 4. Discussion 

Educational actions need to be taken to prepare for old age (the whole society), through old age (for the youngest generation), and in old age (elderly people) [5]. These activities need to account for the attitudes toward this period of life exhibited both by an individual and by the general public. Therefore, it is necessary not only to recognize factors that influence those attitudes, but also to monitor them systematically, because they are not constant values, but constantly fluctuate. The findings of this study proved initiatives aimed at improving an elderly person’s image and social attitudes toward the elderly to be unsatisfactory despite the fact that many such actions have been undertaken. This is because an average of 126.46 was scored in the KAOP scale points, which shows that although the attitude was positive, it was still at a low level. A similar result was obtained from previous research administered in Poland nearly five years ago [6]. There has also been earlier studies that have showed the predominance of neutral attitudes toward the elderly [17]. 

A review of worldwide literature shows a different picture of attitudes toward elderly people. Elderly people are treated with due respect in some countries (e.g., in Thailand) where elderly people, owing to the culture of the country, are treated as a source of life wisdom and are the focal point of every family. Consequently, attitudes toward the elderly are positive within every age group [18]. At the same time, a low level of positive attitudes toward elderly people predominates in China [19], whereas European countries such as Ireland, the UK, and Sweden show decidedly negative attitudes to elderly people. In particular, such negative attitudes have been observed in young generations [6,18,20]. These phenomena frequently stem from elements characteristic of elderly populations in a given country. In 2018, the average life expectation for a male and female newborn baby was 73.8 years and 81.6 years, respectively. Continuing a healthy life is estimated at 8.3 years and 8.6 years for Polish 65-year-old males and females, respectively. For the last 25 years, Poland has seen an over twofold increase in the number of people aged between 60 and 64, while 17.5% of this group were over 80, and the feminization period in the cohort of seniors has decreased by 16% since the 1990s (in 2018, it was 113 for people between 60 and 64, and reached 196 in the group of people between 80 and 84 [5]). However, an increase in the number of elderly people in Poland did not directly translate into improving the perception of this stage of life in Poland. 

Younger people (those under 60) in the researched cohort showed more positive attitudes toward the elderly than people of 60 and older. Similar results were obtained by other researchers who had previously analyzed this phenomenon in Poland [17]. However, different attitudes were adopted by young Americans, as they showed a higher level of negative attitudes toward the elderly [7]. The results of this study did not show an association between respondents’ sex and marital status and their attitudes toward the elderly. Other researchers obtained similar results [6,21,22,23,24,25]. 

Respondents with primary or vocational education exhibited most negative attitudes toward the elderly, whereas those with higher education showed most positive attitudes. This was confirmed in research by other authors [21,26], People with good material situations also scored higher, which means they had more positive attitudes. However, previous investigations administered in Poland fail to confirm this association [6,21]. In our study, the association found may be related to the higher self-esteem showed by the respondents with good material situations.

Respondents obtained the average score of 29.01 on the SES self-esteem scale, which means their self-esteem was at the average or common level. In comparison, Iranian respondents scored higher, as their self-esteem was 35.63 [11]. The result within the research cohort depended on the sex (males sored higher) and the material situation (those with good material situation scored higher). The influence of material situation on the score is confirmed by research findings obtained in Germany [12].

Other analyzed variables did not influence self-esteem. Higher results were obtained by those below 60, married people, and those with higher education. A review study showed similar correlations. A high self-esteem makes it possible to adapt to the growing old process without the feeling of being dispensable, dependent on others, or a source of problems for the close ones [27]. It is also connected with preserving a better physical activity [28,29], and influences the intensity of the fear of death [30]. A low self-esteem may result from the fact that elderly people find it difficult to adjust to a dynamically changing reality (e.g., digitalization), which may lead to them being treated inappropriately or even being discriminated against. 

Respondents with high SES scale results exhibited more positive attitudes toward the elderly. A correlation between self-esteem and perceiving old age and growing old was also confirmed by other researchers [6,21,31].

The authors are aware of research limitations resulting mostly from the insignificant size of the study cohort and the choice of the research tool. Nevertheless, the findings prove it is necessary to constantly monitor the attitudes toward old age and also to more profoundly analyze the factors that might affect such attitudes because even elderly people themselves fail to accept being old. Additionally, such future analyses need to account not only for individual conditions, but also for the influence of sociocultural factors specific for a given population. The research was administered in an area that is not heavily urbanized and has hardly any heavy industry. The family structure has been multigenerational until very recently. On the other hand, this is the area with the fastest increase in the number of elderly people, as well as people over 80, resulting from emigration of the young workforce to other Polish regions and abroad.

## 5. Conclusions

(1) Research respondents perceived old age positively, albeit at a rather low level. It is necessary to review social policies aimed at changing attitudes toward old age.
(2) Actions need to be undertaken aiming at raising self-esteem levels in Polish senior citizens. Moreover, available financial, social and psychological resources from the government and community organizations should all be used to this end.

## Figures and Tables

**Table 1:** Characteristic of the investigated group.

Variable	Variable Category	Numerical Strength (*N*)	Percentage (%)
Age	Below 60	136	66.01
	60 and older	70	33.99
Sex	Female	148	71.84
	Male	58	28.16
Marital status	Single (unmarried; divorcee; widow/widower)	89	43.20
	Married	117	56.80
Education	Primary/vocational	33	16.02
	Secondary	80	38.83
	Higher	93	45.15
Material situation	Good	186	90.29
	Bad	20	9.71

**Table 2:** Respondents’ self-esteem.

Variable	Variable Category	SES		Statistical Analysis	
		M	SD		
Age	Below 60	30.07	4.39	Z = −1.449	*p* = 0.147
	60 and older	29.30	3.92		
Sex	Female	29.70	4.32	Z = −1.997	*p* = 0.046
	Male	32.05	4.36		
Marital status	Married	30.22	4.27	Z = −0.407	*p* = 0.684
	Single	29.91	4.54		
Education	Primary/vocational	29.16	4.36	H = 4.603	*p* = 0.100
	Secondary	29.45	3.94		
	Higher	30.33	4.44		
Material status	Good	30.12	4.08	Z = −2.897	*p* = 0.004
	Bad	26.90	4.71		

SES-Rosenberg Self-Esteem Questionnaire by Morris Rosenberg; Z—U Mann–Whitney test; H—Kruskal–Wallis test; M—median; SD—standard deviation.

**Table 3:** Attitudes within the research cohort toward the elderly.

Variable	Variable Category	KAOP Scale		Statistical Analysis	
		M	SD		
Age	Below 60	127.06	76.00	Z = −0.290	*p* = 0.772
	60 and older	125.34	17.63		
Sex	Female	127.29	18.50	Z = −0.196	*p* = 0.845
	Male	124.40	16.94		
Marital status	Single	124.34	20.40	Z = −1.832	*p* = 0.067
	Married	129.78	15.71		
Education	Primary/vocational	120.79	22.36	H = 5.479	*p* = 0.065
	Secondary	126.40	16.78		
	Higher	128.56	17.22		
Material situation	Good	126.99	18.51	Z = −2.097	*p* = 0.036
	Bad	121.65	12.73		

KAOP—Kogan’s Attitudes toward Old People Scale; Z—U Mann–Whitney test; H—Kruskal–Wallis test; M—median; SD—standard deviation.

## References

[1] Kropińska S., Tobis S., Jakrzewska-Sawińska A., Wieczorowska-Tobis K. (2013). Dlaczego boimy się starości?. Geriatria.

[2] Adamczyk M. (2016). Wizja starości w opiniach Polaków. Studium socjologiczne. Rocz. Nauk Społecznych.

[3] Główny Urząd Statystyczny (2014). Sytuacja Demograficzna Osób Starszych I Konsekwencje Starzenia Się Ludności Polski W Świetle Prognozy Na Lata 2014–2050.

[4] Załącznik do uchwały nr 161 rady ministrów z dnia 26 października 2018 r. (poz. 1169) (2018). Polityka Społeczna Wobec Osób Starszych 2030. Bezpieczeństwo–Uczestnictwo–Solidarność.

[5] (2018). Informacja O Sytuacji Osób Starszych W Polsce Za Rok. https://www.gov.pl/web/rodzina/informacja-o-sytuacji-osob-starszych-w-polsce-za-rok-2018.

[6] Strugała M., Talarska D., Mińska M. (2016). Postrzeganie starości i starzenia się przez osoby dorosłe i w wieku podeszłym. Pielęgniarstwo Pol..

[7] Berger R. (2017). Aging in America: Ageism and General Attitudes toward Growing Old and the Elderly. Open J. Soc. Sci..

[8] Meisner A.B. (2012). A Meta-Analysis of Positive and Negative Age Stereotype Priming Effects on Behavior Among Older Adults. J. Gerontol. Ser. B.

[9] Eiamkanchanalai S., Assarut N., Surasiengsunk S. (2017). Attitude toward the elderly and social interaction: Approach towards an intergenerational society. Kasetsart J. Soc. Sci..

[10] Kilijan M. (2018). Individual and social consequences of old age stereotypes. Forum Pedagog..

[11] Jafari F., Khatony A., Mehrdad M. (2015). Self-esteem, among the elderly visiting the healthcare centers in Kermanshah–Iran. Glob. J. Health Sci..

[12] Orth U., Maes J., Schmitt M. (2015). Self-esteem development across the life span: A longitudinal study with a large sample from Germany. Dev. Psychol..

[13] CBOS (2009). Postawy Wobec Ludzi Starszych I Własnej Starości; Komunikat Z Badań CBOS BS/157/2009.

[14] Łaguna M., Lachowicz-Tabaczek K., Dzwonkowska I. (2007). Skala samooceny SES Morisa Rosenberga-polska adaptacja metody. Psychol. Społeczna.

[15] Kogan N. (1961). Attitudes toward old people: The development of a scale and an examination of correlates. J. Abnorm. Psychol..

[16] Strugała M., Zielińska A., Dymek-Skoczyńska P., Czyżewska-Torba P. (2013). Narzędzia pomiarowe służące ocenie postaw społecznych względem osób starszych- krótka charakterystyka. Now. Lek..

[17] Trempała J., Zając-Lamparska L. (2007). Postawy wobec starszych: różnice międzypokoleniowe. Przegląd Psychol..

[18] Runkawtt V., Gustafsson C., Engström G. (2012). Different Cultures but Similar Postive Attitudes: A Comparison between Thai and Swedish Nursing Students’ Attitudes toward Older People. Educ. Gerontol..

[19] Zhang H., Sun H. (2019). Knowledge, attitude and self-efficacy of elderly caregivers in Chinese nursing homes: A cross—Sectional study in Liaoning Province. BMJ Open.

[20] Gallagher S., Bennett K., Halford J. (2006). A comparison of acute and long-term health-care personnel’s attitudes towards older adults. Int. J. Nurs. Pract..

[21] Strugala M., Talarska D., Wysocki J. (2016). Attitudes towards the Elderly among Nursing Students in Poland—Initial Findings. J. Gerontol. Geriatr. Res..

[22] Wojciszek A., Majda A., Nawalana A. (2013). Postawy ludzi uzupełniających wykształcenie średnie wobec osób starszych. Probl. Pielęgniarstwa.

[23] Lagace M., Charmarkeh H., Grandena F. (2012). Cultural Perceptions of Aging: The Perspective of Somali Canadians in Ottawa. J. Cross Cult. Gerontol..

[24] Allen K.R., Roberto K.A. (2009). From Sexism to Sexy: Challenging Young Adults’ Ageism about Older Women’s Sexuality. Sex. Res. Soc. Policy.

[25] D’Souza A.F., Sunny A.M., Thomas J.M., Sequera S.K.L. (2018). Attitude of the middle aged adults towards Elderly population at selected setting, Mangaluru. Asian J. Nurs. Educ. Res..

[26] Luchesi B., Alexandre T., De Oliveira N., Brigola A., Kusumota L., Pavarini S., Marques S. (2016). Factors associated with attitudes toward the elderly in a sample of elderly caregivers. Int. Psychogeriatr..

[27] Lang F., Heckhausen J. (2001). Perceived Control Over Development and Subjective Well-Being: Differential Benefits Across Adulthood. J. Personal. Soc. Psychol..

[28] Jodra P., Luis Maté-Muñoz J., Domínguez R. (2019). Perception of health, self-esteem and physical self-concept in elderly persons based on their physical activity. Rev. Psicol. Deporte.

[29] Mora-García J.E., Orgaz García D., López García S., Amatria Jiménez M., Maneiro Dios R. (2018). Influence of physical activity on self-esteem and risk of dependence in active and sedentary elderly people. An. Psicol..

[30] Zhang J., Peng J., Gao P., Huang H., Cao Y., Zheng L., Miao D. (2019). Relationship between meaning in life and death anxiety in the elderly: Self-esteem as a mediator. BMC Geriatr..

[31] Mossakowska M., Więcek A., Błędowski P. (2012). Aspekty Medyczne, Psychologiczne, Socjologiczne I Ekonomiczne Starzenia Się Ludzi W Polsce.

